# Qualitative study of the feasibility of HPV vaccine delivery to young adolescent girls in Vietnam: evidence from a government-implemented demonstration program

**DOI:** 10.1186/1471-2458-14-556

**Published:** 2014-06-05

**Authors:** D Scott LaMontagne, Nguyen Quy Nghi, Le Thi Nga, Amynah Janmohamed, Dang Thi Thanh Huyen, Nguyen Tran Hien, Vivien Davis Tsu

**Affiliations:** 1 Vaccine Access & Delivery Department, PATH, PO Box 900922, Seattle, WA 98109, USA; 2 World Bank, 8th Floor, 63 Ly Thai To, Hanoi, Vietnam; 3 HealthBridge, Suite 202&203, E4 Building, Trung Tu Diplomatic Compound, No.6 Dang Van Ngu, DongDa, Hanoi, Vietnam; 4 Faculty of Land and Food Systems, University of British Columbia, 2357 Main Mall, Vancouver, BC V6T1Z4, Canada; 5 National Expanded Program on Immunization, National Institute of Hygiene and Epidemiology, 1 Yersin St, Hai Ba Trung District, Hanoi, Vietnam; 6 Reproductive Health Global Program, PATH, PO Box 900922, Seattle, WA 98109, USA

**Keywords:** HPV vaccine, Feasibility, Health system, School-based, Vaccine delivery, Vietnam

## Abstract

**Background:**

Introduction of human papillomavirus (HPV) vaccine in national programs has proceeded apace since 2006, mostly in high-income countries. Recently concluded pilots of HPV vaccination in low-income countries have provided important lessons learned for these settings; however, rigorous evaluations of the feasibility of these delivery strategies that effectively reach young adolescents have been few. This paper presents results from a qualitative evaluation of a demonstration program which implemented school-based and health center–based HPV vaccinations to all girls in grade 6, or 11 years of age, for two years in four districts of Vietnam.

**Methods:**

Using semi-structured interviews of 131 health and education staff from local, district, province, and national levels and 26 focus-group discussions with local project implementers (n = 153), we conducted a qualitative two-year evaluation to measure the impact of HPV vaccinations on the health and education systems.

**Results:**

HPV vaccine delivery at schools or health centers was made feasible by: a. close collaboration between the health and education sectors, b. detailed planning for implementation, c. clearly defined roles and responsibilities for project implementers, d. effective management and supervision of vaccinations during delivery, and e. engagement with community organizations for support. Both the health and education systems were temporarily challenged with the extra workload, but the disruptions were short-lived (a few days for each of three doses) and perceived as worth the longer-term benefit of cervical cancer prevention.

**Conclusion:**

The learning from Vietnam has identified critical elements for successful vaccine delivery that can provide a model for other countries to consider during their planning of national rollout of HPV vaccine.

## Background

Safe and effective human papillomavirus (HPV) vaccines provide a new opportunity for cervical cancer primary prevention [1,2]. National introduction of HPV vaccine has proceeded apace since 2006, with vaccinations to occur in 54 national programs by the middle of 2013 [3,4]. Initial introduction from 2006 to 2008 was largely contained in high-income countries, such as Australia, the United States, and Europe, where the rates of cervical cancer are lowest [4,5]. Since 2008, a handful of middle-income countries, such as Mexico and Panama, have phased in HPV vaccine with purchases at discounted prices through the Pan-American Health Organization (PAHO) Revolving Fund [6]. Rwanda became one of the first low-income countries to introduce HPV vaccine nationally in 2011, representing a new phase in the global adoption of this life-saving intervention [7].

Much of the post-introduction literature has come from high-income countries, with a focus on coverage levels achieved, provider acceptability, and early impact of vaccination on disease endpoints [8-15]. However, there is a dearth of literature evaluating the mechanics of delivery and feasibility of the health system to incorporate HPV vaccine [16,17]. Experiences from implementation in high-income countries may not necessarily be relevant or translatable to low-income countries, which have the most to gain from adoption of HPV vaccine, given that more than 88 percent of all deaths from cervical cancer occur in these countries [18].

On a small scale, some evidence of the feasibility of HPV vaccine delivery in developing countries is emerging from the evaluation of pilot projects or demonstration programs, which have provided insight into both the challenges of implementation and the components of the programs that have been successful [7,19,20]. A large demonstration program of school-based HPV vaccine delivery in Peru found that close coordination with the schools was key for successful delivery [19], and the national introduction of HPV vaccine in Rwanda noted the strong political support from the national to the community level as being critical to vaccine uptake [7]. Conversely, a review of eight pilots done through small donations of HPV vaccine suggested that insufficient training and sensitization of teachers and school headmasters, lack of community participation, and loss-to-follow-up can be significant barriers for feasible programs [20].

Examples of HPV vaccine implementation within the routine immunization systems of Asian countries are largely absent. Formative research, prior to vaccine availability, from India and Vietnam, explored the types of models for HPV vaccine delivery that communities would accept and that governments would find feasible [21-24]. These studies found a strong immunization delivery system in place, as well as communities and policymakers receptive to the idea of a vaccine to prevent cervical cancer [22-24]. The government of Vietnam utilized the results from this formative research to design and implement a two-year demonstration project to test whether a school-based or health center–based delivery of HPV vaccine would result in higher coverage, achieve strong community acceptance, and be feasible within the existing health system [25]. This project was implemented in 72 communes of four districts in Thanh Hoa province (rural, in the north) and Can Tho city (urban, in the south) in Vietnam by the National Expanded Program on Immunization (NEPI) under the auspices of the National Institute of Hygiene and Epidemiology (NIHE) and in collaboration with the National Center for Health Education and Communication (NCHEC) and PATH [26]. All girls in grade 6 regardless of age (school-based) or 11 years of age (health center–based) were targeted, with vaccinations occurring a day before or a day after the regularly scheduled monthly date for routine immunizations of infants. Delivery was pulsed: one day allocated for delivery of each of three doses of HPV vaccine [25], using the standard schedule of 0, 2, 6 months.

The HPV vaccine demonstration project implemented in Vietnam in 2009 and 2010 was evaluated for coverage achieved by the strategies, community acceptability, system feasibility, and incremental economic and financial costs of delivery. Project details and results for coverage achieved, acceptability by parents, and costs of implementation have been published elsewhere [25-27]. However, to briefly summarize, coverage in the first year was 83% in school-based delivery and 94% for health-center based delivery, and for year two coverage was 96% and 99%, respectively [25]. The reasons for the lower coverage in the first year for school-based delivery was systematically evaluated and related to acceptability, with rumors on safety influencing parental decisions in one urban district only [25,26]. Coverage in the other three project districts in the first year that delivered vaccine via schools was similar to that achieved through the health center-based approach. Using reporting guidelines for qualitative studies (RATS), this paper presents the findings from a two-year qualitative evaluation of the feasibility of HPV vaccine delivery to determine the impact of incorporating HPV vaccine on the health and education systems in Vietnam.

## Methods

### Study design and objective

We designed a descriptive qualitative study to determine the impact of incorporating HPV vaccine on the five levels of the health and education system in Vietnam: national, regional, provincial, district, and commune.

### Study population, sampling frame, and sample size

We employed criteria-based purposive sampling to select 24 communes for study inclusion. All 72 communes participating in the demonstration program were divided based on HPV vaccine-delivery strategy (school-based or health center–based), geographic stratum (mountainous, rural, and urban), and first dose HPV vaccine uptake rate (low versus high). After categorization, 12 communes were randomly selected in each year and represented both delivery strategies (6 school-based and 6 health-center programs), all three geographic strata (4 urban, 4 rural, 4 mountainous), and high and low uptake (6 high and low). All 72 communes were randomized in each year so any individual commune had a chance to be selected into both the year 1 and year 2 samples: 3 of the final 24 selected communes participated in the study in both years. Within each commune, specific populations were purposively selected based on their involvement with delivering HPV vaccinations. These included health workers, teachers, community leaders, and other key officials or stakeholders. In the Vietnamese context, each commune has one health center and usually one school, so participants in the HPV vaccine delivery program were known and invited to voluntarily participate by the research team. Both communes and individual study populations within communes were selected to provide breadth of programmatic experience, a technique common for qualitative research [28,29], and not for sample representativeness, as is done in a quantitative study.

After the first round of vaccinations was complete, 112 semi-structured interviews (SSIs) with NEPI and NCHEC national and regional leaders, government leaders, health and education staff at provincial, district and commune levels were completed; after the second year of implementation, 26 focus group discussions (FGDs) with 153 project implementers and an additional 19 semi-structured interviews with national, regional, and provincial leaders in the health system were conducted (Table 1). We utilized SSIs in year 1, as it was not yet known how comfortable program implementers would be in discussing operational aspects that may be considered sensitive. However, results from year 1 illustrated high levels of collaboration between program implementers, reflecting the trusting working relationship; thus, in year 2, FGDs were utilized for primary data collection.

**Table 1:** Study populations, sample size, and data collection methods, feasibility evaluation of HPV vaccine delivery, Vietnam, 2009-2010

**Study population**	**Data collection technique**	**Year 1**	**Year 2**		
		**No. of participants**	**No. of sessions**	**No. of participants**	**Total**
Community leaders/health managers	SSI	8	n/a	9	17
Health workers, community mobilizers, teachers	SSI	104	n/a	10	114
	FGD	n/a	26	153	153
Total		112		172	284

*Abbreviations*: *FGD* Focus Group Discussion, *SSI* Semi-structured Interview.

### Data collection and analysis

All interviews and FGDs were conducted by trained researchers^a^ and based on a question guide, covering areas of inquiry relevant to the objective of the feasibility evaluation and covered major operational areas of vaccine delivery systems required for all vaccines, including HPV [17,19,20,22,25]. To assess the impact on the health system, questions related to human resources, workload, system constraints, vaccine storage, transport, and cold chain were asked. Questions specific to the school setting—such as preparations, involvement of school staff, disruptions, and coordination with the health sector—were asked to reflect the impact on the education system. An overarching theme of collaboration between sectors and across the four principal levels of the Vietnamese health system (commune, district, provincial, and national) was probed through an additional series of questions. For all respondents, other areas of inquiry included: health-facility services, personnel, and working hours; adequacy of preparations for community sensitization and mobilization; process of enumerating vaccine-eligible girls prior to vaccination and efforts taken to reach girls not in school; and activities that should or should not be replicated for future vaccinations—i.e., what worked well and what worked less well.

All conversations were tape-recorded and written in researchers’ field notes. Interviews and FGDs took place in a setting conducive to ensuring privacy, most often at either health centers or schools nearby health centers. We created a qualitative data repository system, including an electronic database, to facilitate organization, security, quality control, maintenance, and analysis of study data. All data were cleaned, coded, and de-identified prior to analysis. Field notes were entered into Atlas.ti software (ATLAS.ti Scientific Software Development, Berlin, Germany) for data management, after verification of accuracy with tape recordings of interviews and FGDs. All qualitative data from SSIs and FGDs were then transferred into NVivo software (QSR International, Burlington, MA, USA) for analysis using a qualitative, thematic approach [28], which involved sorting, categorizing, and summarizing data to answer questions framed by each key area of inquiry (outlined above). All results from SSIs and FGDs were grouped according to three major themes – impact on the health system, impact on the education system, and intersectoral collaboration. Direct quotes from study participants are presented in the results section as representations of the most salient and prevalent themes and subthemes identified from all interviews and FGDs—a data presentation technique common in qualitative research [29]. All data were collected and analyzed in Vietnamese. However, for quality control and data reporting, English translations and back translations of thematic analyses, especially for the quotes were conducted by two co-authors (NQN and LTN). Two co-authors who are native English speakers (DSL and AJ) verified the English translation of the thematic analyses.

### Ethics

Verbal consent was solicited from all study participants using a pre-defined script. A member of the research team involved in either the interview or FGD noted by signature on the data recording form that verbal consent was received. All respondents were given a small thank-you gift (value less than US $1.00) as a token of appreciation, per Vietnamese custom. The research protocol was approved by the internal review board of NIHE (Vietnam) and the research ethics committee of PATH (USA).

## Results

### Impact of HPV vaccinations on the health system

HPV vaccine introduction impacted personnel and the regular activities of commune health centers in several ways. Shortage of personnel on vaccination day was mentioned after both rounds of vaccinations. To deal with this problem, many commune health centers mobilized additional personnel from commune-level mass organizations or health staff from districts and provinces.

*We usually mobilized staff from reproductive health units, disease control units, or food safety and security units for not only HPV vaccination but also for monthly EPI* [Expanded Program on Immunization] *activities at all 13 communes from the first to the fifth. We did not have adequate staff.*

SSI, District health staff, School-based strategy, Can Tho city, urban

The first sessions of HPV vaccination needed the support from the People’s Committees, educational sector, and civic organizations such as the Women’s Union. Health officials took the lead in garnering support from these organizations, while also being responsible for conducting vaccinations. During the first round of vaccinations delivery of HPV vaccine with existing health worker staff created extra work. However, this was reduced in the second round, with only one mountainous commune particularly affected.

*As we have few health workers, the arrangement of one-way flow with many tables for vaccination* [4 stations: screening, counseling, vaccinating, recording]*, sessions are sometimes difficult for us. Due to the lack of staff, we also have more difficulties in doing both communication and vaccination activities than in other places.*

FGD, Health workers in Nam Xuan and Hien Kiet communes, Quan Hoa district, Thanh Hoa province, mountainous

Health staff emphasized that introduction of a new vaccine required greater investment in communication activities and training to deliver the first dose, leading to significant changes in their workload, which decreased for the second and third doses. Additionally, during vaccination sessions, standard procedures and the process of immunization were followed according to project guidelines, which required more staff for screening and counseling girls in comparison to other immunization days (for children and infants). The project also required more supervision, which placed a greater burden of work on health staff in provinces and districts.

Communicators at villages were involved in communication. District reproductive health staff also supported us in training communicators. During vaccination days, we ourselves divided into small teams to go to communes. As this vaccine is new, communication to parents was conducted in the school, but several people still did not understand well. Thus, we must visit families to explain more.

SSI, District health staff, Health center–based strategy, Can Tho city, urban

Though faced with a heavy workload, health workers acknowledged that the situation was under control and workable. To accomplish the task with their limited resources, they developed a reasonable working calendar. They also showed commitment to the work, given the benefits of the vaccine for the community.

We should save time for this activity. Although sometimes it overlapped our work and schedule and caused difficulty for us, with our determination, endeavor, and enthusiasm, along with direction from higher level, we tried to complete the task.

FGD, Health worker in Hung Loi and An Cu communes, Ninh Kieu district, Can Tho city, urban

Sentiments from commune and district-level health workers were reinforced by interview data from government and health leaders. Most leaders confirmed that “staff were working at their maximum capacity.” Leaders from both government and the health sector also mentioned that district and commune staff were able to arrange to have enough staff for the vaccination sessions. There was no need to recruit new staff for HPV vaccination.

Identifying girls eligible for HPV vaccination was noted by health workers as a task that was time-consuming and contributed to the heavy workload. Making a list of eligible girls for HPV vaccinations is considered a critical activity in the HPV vaccination program that requires precise supervision, as this list is used for vaccine and other equipment supplies. In the communes that used school-based delivery, the list was first taken from schools and reviewed by commune health staff. In communes with health center–based delivery, the list was obtained from the village first and then compared with that from the schools. We found little difference in the burden of this task between the two strategies, since the list was collected from two sources, in either case, and checked by health staff for the final version.

On the whole, however, most study participants from district and commune levels, regardless of delivery strategy utilized, reported that routine health services were not negatively impacted by HPV vaccine delivery. After the first round of vaccinations, 61.8 percent of health workers and 78.3 percent of community leaders who were interviewed reported that HPV vaccination did not affect routine health services or activities. They attributed this to health workers being familiar with immunization activities, enabling them to prioritize their time and work during HPV vaccination days, the extra assistance from non-health personnel (leaders, teachers, etc.), and the benefit of vaccination sessions not lasting too long (taking up one morning for administration of each dose). Immunization program staff at the provincial level responded similarly.

The densely populated district in the south that participated in the HPV vaccine demonstration project did, however, report negative impacts of HPV vaccine introduction on regular activities of the commune health center, such as reduced services for health checks and treatment during the vaccination session. They reported that more time and energy of health staff were put into preparation activities, including training, planning meetings, listing eligible girls, and organizing communication activities, and these activities affected their other health activities.

In both provinces, HPV vaccines were transported along administrative levels from province to district by specialized car and/or cold boxes and from district to commune by motorcycle and vaccine carriers. Health workers did not report any difficulty in the transport and storage procedures of HPV vaccines in the program, which followed guidelines from the Ministry of Health. At the provincial level, the cold chain was equipped with sufficient capacity in terms of storing and transporting vaccine to lower levels.

Most participants said that vaccine had been transported and stored following the current effective regulations and under close supervision. There were no complaints about cold chain capacity for vaccine storage or that the addition of HPV vaccines affected the storage and cold chain of the other routine vaccines in the participating districts.

*No effect. We have three cold boxes, then there is no problem if we integrate* [HPV vaccination] *into routine immunization… Every month we have only about 30 children for routine vaccination. For HPV vaccine it depends on age groups.*

SSI, Leader of District Health Center, Thanh Hoa province, rural

### Impact of HPV vaccination program on the education system

The involvement of schools was more intensive in the school-based strategy due to vaccinations occurring while girls were in school. From the education system perspective, the most frequently cited effects included impacts on the learning activities of students, school activities, and the volume of work for teachers. Some schools arranged vaccination days during the school day when all girls in grade 6 were allowed to leave class for about 45-50 minutes to get vaccinated in a separate room which was set-up for this purpose. Some schools organized vaccination days after school hours, but vaccinations were still conducted on the school premises. Most schools had more than one class of girls in grade 6, so multiple vaccinations sessions were run on the day of vaccination.

Learning activities of pupils were the most influenced when vaccination was organized during workdays, regardless of the strategy. In general, pupils needed about 45 minutes to complete the vaccination process.

*First, as this vaccination should be completed in the morning, we decided to organize immunization sessions in the morning. Thus, all children left class whenever health workers came. After injection, children could not continue to study, so we decided to close the class at that time. Children who had missed lessons should come in the afternoon next day. Thus, it is only* [a]*delay of* [the] *school day,* [and] *school week, but not* [a] *delay of* [the]*yearly program. So, besides working in that morning (immunization session), teachers must work one more afternoon.*

SSI, Vice Headmaster, School-based strategy, Thanh Hoa province, rural

The impact on learning and teaching activities depended on school coordination capacity. Many schools had very good coordination, inviting students from the same class to a waiting room, then taking them to a vaccination room individually to minimize the disruption and effects on other classes.

*We had three tables for injection. It took only 20 minutes for one class. At that time, boys went to* [the]*library for reading books. So, children did not miss class, as we planned very well. We got support from* [the] *management board and parents of children.*

FGD, Teacher, School-based strategy, Can Tho city, urban

Due to the shortage of physical space, many schools mobilized meeting rooms, libraries, etc., for vaccination days. On vaccination days, activities in these locations were significantly disrupted. Additionally, teachers noted that the HPV vaccine delivery schedule needs to be coordinated with the examination schedule for students, “… *if the vaccination session falls in with examination day, then the school needs to change its schedule to not influence results of the students”.* Some participants mentioned that doing HPV vaccine introduction in the school increases the workload of teachers, notably head teachers and/or school health officers.

On vaccination day, eligible school girls had to be absent from class for half an hour or an hour, it affected the training and learning schedule of teacher and girls, so the school authority had to arrange another schedule for them in return for this absence.

FGD, Health workers in Hung Loi and An Cu communes, NinhKieu district, Can Tho city, urban

However, most interviewed teachers considered vaccine delivery in schools to cause minimal disruption and that prevention of cervical cancer was worth the disruption and inconvenience of school lessons.

### Intersectoral collaboration

Collaboration across sectors was a major theme reiterated by most respondents as being critically important to the success of the HPV vaccinations implemented. The example of collaboration was set at the national level and reinforced by provincial and district-level collaboration between the health and education sectors, all of which created a supportive environment for health and education collaborations at the lowest level of the communes.

At the provincial level, the collaboration between the health and education sectors focused more on broader issues, such as policies to facilitate project implementation at lower levels. The health sector played the key implementation role, while the education sector provided support.

*Health and education sectors cooperated closely and effectively during the implementation of* [the] *HPV vaccine delivery project. Both sectors agreed on the activity plan and communication content.*

SSI, Director, Provincial Center for Health Education and Communication, Can Tho city, urban

Meetings were held by the two sectors to agree on a joint document that clearly outlined the roles and responsibilities of each party in implementing HPV vaccinations. At district and commune levels, local authorities were more directly involved in project activities. Study participants credited the direction and participation of local authorities for the favorable support received from different departments and organizations.

*It* [the People’s Committee] *helped to mobilize and coordinate the participation of different departments and organizations in the HPV project.*

SSI, Director, District Preventive Medicine Center, Nong Cong district, Thanh Hoa province, rural

The education departments within targeted districts directly participated in certain activities as members of project coordination boards at the district level. This management scheme helped education leaders have a broad view of the project design and activity plans, allowing them to offer timely direction to schools in conducting scheduled activities.

At the commune level, the collaboration involved joint implementation of project activities. Respondents reported that health centers were the responsible contact, directing all project activities at communes, with schools participating in preparation, communication activities, and vaccination sessions. School activities included: a) preparing lists of eligible girls; b) organizing parent meetings at schools; c) conducting communication sessions for students and parents, which included inviting health officials to speak; d) delivering invitation letters and reminding girls of the vaccination day; and e) participating jointly on vaccination days.

For school-based delivery, schools participated in preparing the location for vaccinations, facilitating the participation of eligible girls, and managing girls during and after vaccination. For health center–based delivery, schools facilitated the participation of eligible girls by allowing them to take a day or a session off during vaccination day, if it was organized during class time. But this occurred only three times over the entire school year, once for each dose, so missed lessons were easily made up on other days. In some communes, teachers escorted the girls to receive vaccinations.

Local project implementers reported that the People’s Committee leaders not only mobilized the participation of other organizations, but also directly launched the first dose of the HPV vaccination campaign and actively supervised and encouraged project implementers during those vaccination days. Under the direction of local authorities, many other organizations, such as cultural and information departments, Women’s Unions, and Youth Unions, also participated in project implementation. Respondents suggested that, like other development projects, the participation of local authorities in coordinating project activities for HPV vaccinations facilitated community acceptability and participation of the health and education sectors.

## Discussion

At the advent of HPV vaccine availability, commentators and researchers contemplated the difficulties of implementation, especially for developing countries, because: a) the vaccine targeted an age group not typical for immunization programs; b) the disease and HPV virus as the cause were not well known; c) the dosing schedule required three shots over six months; and d) the resources needed for sensitization, mobilization, and implementation could overwhelm the health system [30-33]. Despite these issues, many countries proceeded with recommendations for vaccination [5] and organized programs began to be implemented [4], some showing early signs of success in reaching adolescents as measured by first-dose uptake and three-dose coverage [16,34]. However, as Binagwaho and colleagues note, there was resistance for developing countries to implement HPV vaccine because international organizations felt that resources might be shifted from other effective child health interventions [35].

Formative research in Vietnam prior to the availability of HPV vaccine also revealed concerns from policymakers about the challenges to be overcome [22,24]. Rather than assuming HPV vaccine delivery was impossible and doing nothing, clear, rational advice and suggestions were given for a delivery strategy that could be feasible [24]. Our study has demonstrated that successful HPV vaccine implementation using a variety of approaches is possible in a low-resource setting like Vietnam. Our findings highlight positive aspects that worked well to support feasible implementation, as has been found in other studies. The program’s credibility with communities is enhanced by visible endorsement by national and local government officials and key stakeholders [4,7,19,26,36], and utilization of the existing infrastructure and resources of the national routine immunization program leveraged existing competencies and capacities [7,19,24,36]. As was found in programs implemented in Rwanda, Peru, and Australia, delineated roles and responsibilities for all implementing partners and preparing communities in advance through awareness raising and sensitization were critical aspects to the feasibility of HPV vaccine delivery [7,17,19]. Ladner and colleagues, in their review of pilot programs from Bhutan, Bolivia, Cameroon, Haiti, Lesotho, and Nepal, also emphasized the importance of informing communities about HPV vaccination prior to program implementation [20]. They found effective and intimate collaboration between the education and health sectors to be critical, especially for school-based delivery programs [20]—key learning from both the Peru and Australia programs, as well [17,19]. Factors for successful delivery were remarkably similar for low-, middle-, and high-income countries, providing a roadmap for other countries currently considering how to design their national HPV vaccination strategy. Encouragingly, no notable challenges with vaccine storage, transport and cold chain were found in this study of HPV vaccine delivery in Vietnam [20].

As with any new program, good planning and preparation helps to mitigate significant barriers, but sometimes not everything goes according to plan. As with the successful components of implementation, the negative experiences and challenges experienced by Vietnam were similar to those reported by other countries. For example, generating a list of eligible girls and selecting those who are eligible for the program based on pre-defined criteria was difficult in Rwanda and Peru [7,19], and both Uganda and Tanzania found that school-based delivery is easier if girls are selected according to their grade, rather than by their age [25,36]. Tracking girls for all three doses and coordinating the dosing schedule with the school calendar have also been noted to be difficult for others [7,16,19,20]. Most programs have reported an increase in the workload for health workers to implement HPV vaccinations, but nearly all reported that this situation was temporary, short-lived, did not affect routine services significantly, and could be managed well if careful planning was done [17,19,37]. Binagwaho and colleagues noted that having six fixed days for each of the three doses of HPV vaccine facilitated high coverage, as it was easier to mobilize communities, coordinate activities, and commandeer resources because everyone was aware of what would happen and when [7]. We found that this pulsed approach was also used for HPV vaccine delivery in Vietnam. Studies from Peru and eight other pilots noted that multiple visits to schools and continuously following up with girls was challenging [19,20]. Focusing the vaccinations on three fixed time points, similar to a pulsed campaign delivery, might reduce the overall workload, as such a schedule would bring predictability for communities, schools, and the health system, and it would only temporarily stress other services for just a few days a year. In summary, most of the challenges for the feasibility of HPV vaccine delivery found in our study were logistical, commonly found in a diversity of other HPV vaccination programs, and they were overcome through additional coordination and planning in subsequent rounds of vaccinations.

## Limitations

The limitations of this qualitative study should be noted. The methodology and sampling frame, while rigorous, could still have excluded important perspectives in understanding the layered dynamics of implementing vaccinations to this age group both in schools and through the regularly established health centers. Data collection occurred shortly after the third dose of vaccine was delivered, but recall of earlier activities and experiences by study participants may have introduced unintentional bias. Because of the complex nature of both the delivery of this new vaccine and the overall evaluation design, results from this feasibility study need to be contextualized within the results of other evaluation components already published [25-27].

## Conclusions

This study demonstrated that the feasibility of delivering HPV vaccinations through schools or health centers in Vietnam was accomplished through: a) close collaboration between education and health sectors at national, provincial, district, and commune levels; b) a manageable project plan; c) assignment of clear roles and responsibilities among the parties involved; d) close coordination and supervision during vaccine implementation; and e) promotion of an overall enabling environment by engagement with critical community leadership organizations and health education centers.

## Endnote

^a^Two of the co-authors (NQN and LTN) were part of the interviewing team.

## Competing interests

The authors declare they have no competing interests.

## Authors’ contributions

DSL managed the overall evaluation, designed the study, reviewed, synthesized, and interpreted preliminary results, substantively revised the initial draft of the manuscript, and led on the final revised manuscript. NQN led the study in-country, designed the study, collected and analyzed data, provided initial synthesis and interpretation, including translations and back-translations, drafted the initial manuscript, and reviewed and commented on the final revised manuscript. LTN managed the overall evaluation in-country, designed the study, collected and analyzed data, including translations and back-translations, reviewed, synthesized, and interpreted preliminary results, and reviewed and commented on the final revised manuscript. AJ designed the study, drafted data collection instruments, coordinated ethics reviews, reviewed, synthesized, and interpreted preliminary results, and reviewed and commented on the final revised manuscript. DTTH was responsible for the vaccine implementation in the study districts in Vietnam, provided input into the study design, reviewed preliminary results, and commented on the final revised manuscript. NTH was the senior person in-charge of the vaccine implementation program in Vietnam, provided strategic leadership, reviewed and contributed to the study design, and commented on drafts of the results. VDT was the overall project manager for the PATH four-country HPV vaccine project, commented on study design, reviewed preliminary results, and reviewed and commented on drafts of the initial and final manuscript. All authors read and approved the final manuscript.

## Authors’ information

Nguyen Quy Nghi, Le Thi Nga and Amynah Janmohamed were employed by PATH at the time of this research.

## Pre-publication history

The pre-publication history for this paper can be accessed here:

http://www.biomedcentral.com/1471-2458/14/556/prepub
